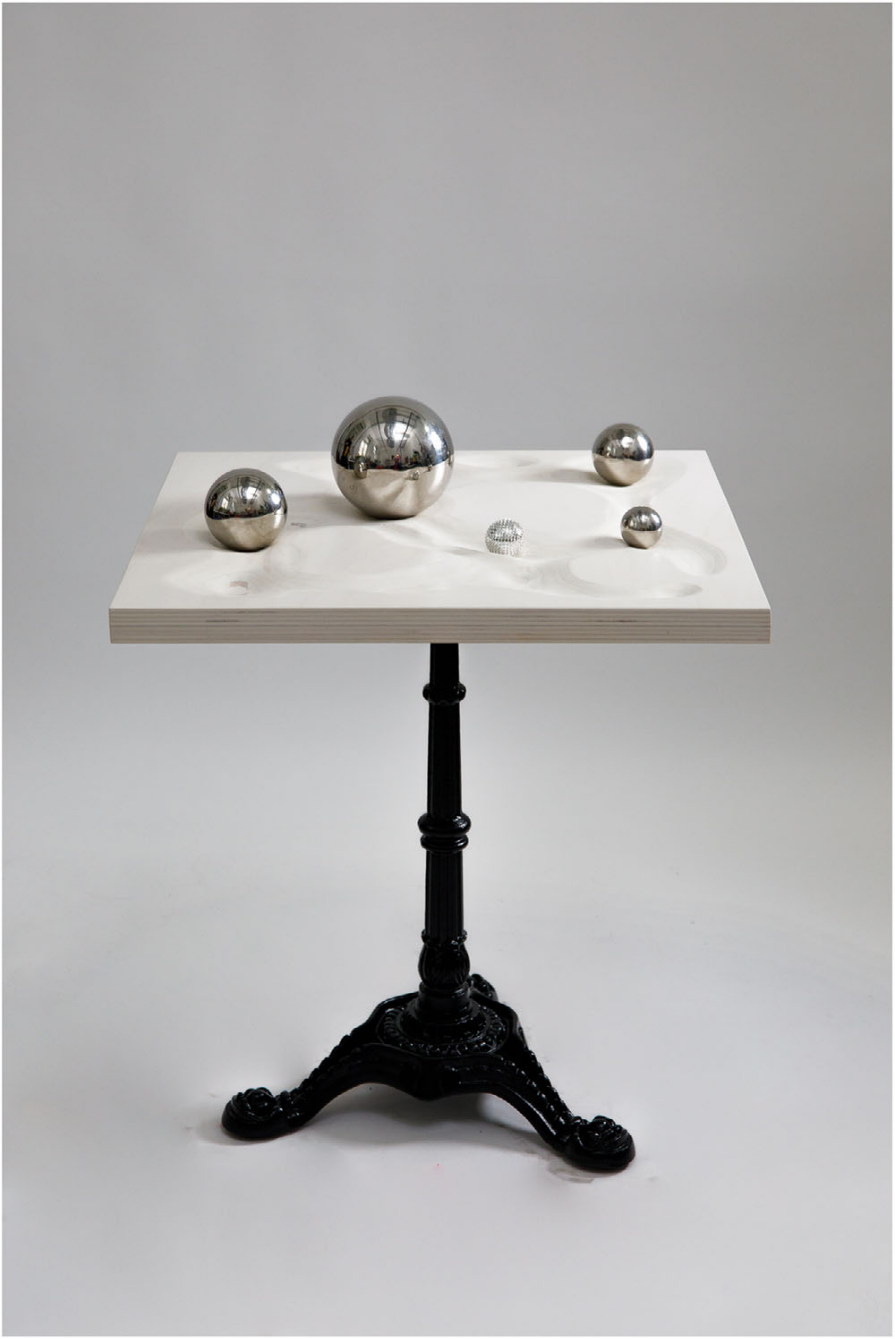# NV23non‐verbal communication

**DOI:** 10.1002/alz.089724

**Published:** 2025-01-09

**Authors:** Pia Moana Scharler‐Plotnik, Demedarts

**Affiliations:** ^1^ University of Applied Arts Vienna, Vienna, Vianna Austria

## Abstract

Imagine a visit in the care taking home by the grandchild. The communication might be challanging. What if there was an option that offers a different way to interact without language? The NV23 is a table designed for non‐verbal and cross generational communication. Sensoric play without the possibility of doing it wrong, as there are no rules of usage. The NV23 has a surface of a landscape and can be filled with various objects. Objects should be selected by the haptic memory, to trigger positiv associations, but could simply be chosen by the appealing shape or color. Everyone who sits at the NV23 can choose or bring objects to the table. The curation of the object depends on the people using it, as well as the use of it. We have experienced ball games, sound checks of materials, storytelling to the different characteristics of the objects in the landscape, and many more. The artistic table have been designed and tested that encourage people with very advanced dementia to interact. The NV23 object has designed in different versions of appearances to test if this also has influence and make it appealing. It is still in development, so the session is an open conversation, a testing and a showing of findings. Pia Scharler is member of the DEMEDARTS project team, which is a artistic‐research team in Vienna.